# Supplementary material for: The genome sequence of the black scavenger fly, Nemopoda nitidula (Fallén, 1820)
Source: Wellcome Open Res. 2025 Feb 7;10:49. [Version 1] doi: 10.12688/wellcomeopenres.23664.1 (PMC12163368; doi:10.12688/wellcomeopenres.23664.1)
Supplement: Peer review history

# Reviewer report: Reviewer response for version 1

**DOI:** 10.21956/wellcomeopenres.26106.r125216

**Published:** 2025-06-12

**Authors:** Thomas Brown

**Affiliations:** 1 Leibniz IZW (Berlin) & ERGA/BGE, Leibniz, Germany

Falk et al present the genome assembly for the black scavenger fly Nemopoda nitudula as part of the DToL program. The methods used are state-of-the-art and well explained in general. A little more explanation could be given as to what features about the phylogeny and evolution of "different traits" that will be uncovered with the genome would give the background section more weight.

 I only have a couple of minor points:

 The abstract states the assembly is from a single specimen, yet the PacBio and Hi-C reads are stated as coming from different specimens in the methods. I find this a little misleading.

 The kmer completeness is stated as 100% complete in the table. I think it should be made clearer that this corresponds to the combination of primary and alternate assemblies, as these are each stated as having completenesses of 76% in the text. The contig and scaffold N50s and BUSCO scores most likely correspond to the primary assembly, I find this misleading

Are sufficient details of methods and materials provided to allow replication by others?

Yes

Is the rationale for creating the dataset(s) clearly described?

Partly

Are the datasets clearly presented in a useable and accessible format?

Yes

Are the protocols appropriate and is the work technically sound?

Yes

Reviewer Expertise:

Genome assembly, Genome annotation, Workflows, FAIR research

I confirm that I have read this submission and believe that I have an appropriate level of expertise to confirm that it is of an acceptable scientific standard.

# Reviewer report: Reviewer response for version 1

**DOI:** 10.21956/wellcomeopenres.26106.r122339

**Published:** 2025-06-06

**Authors:** Terrence Sylvester

**Affiliations:** 1 University of memphis, memphis, USA

The authors provide a high-quality genome assembly of the black scavenger fly, *Nemopoda nitidula. *Genome quality matrices indicate that the presented genome was assembled and curated to minimise assembly errors.  This genome would be a valuable community resource for other researchers. If the authors can include the metrics of the successful extraction that was used for PacBio sequencing (e.g., Nanodrop graph, femtopulse fragment size distribution, etc.), that would be something of value for other researchers, giving a point of reference to compare their projects with. Other than that, I do not have significant remarks on the paper.

Are sufficient details of methods and materials provided to allow replication by others?

Yes

Is the rationale for creating the dataset(s) clearly described?

Yes

Are the datasets clearly presented in a useable and accessible format?

Yes

Are the protocols appropriate and is the work technically sound?

Yes

Reviewer Expertise:

Genomics

I confirm that I have read this submission and believe that I have an appropriate level of expertise to confirm that it is of an acceptable scientific standard.
